# The decomposition of fine and coarse roots: their global patterns and controlling factors

**DOI:** 10.1038/srep09940

**Published:** 2015-05-05

**Authors:** Xinyue Zhang, Wei Wang

**Affiliations:** 1 Department of Ecology, College of Urban and Environmental Sciences, Peking University, Beijing 100871, China; 2 Shenzhen Graduate School, Peking University, Shenzhen 518055, China

## Abstract

Fine root decomposition represents a large carbon (C) cost to plants, and serves as a potential soil C source, as well as a substantial proportion of net primary productivity. Coarse roots differ markedly from fine roots in morphology, nutrient concentrations, functions, and decomposition mechanisms. Still poorly understood is whether a consistent global pattern exists between the decomposition of fine (<2 mm root diameter) and coarse (≥2 mm) roots. A comprehensive terrestrial root decomposition dataset, including 530 observations from 71 sampling sites, was thus used to compare global patterns of decomposition of fine and coarse roots. Fine roots decomposed significantly faster than coarse roots in middle latitude areas, but their decomposition in low latitude regions was not significantly different from that of coarse roots. Coarse root decomposition showed more dependence on climate, especially mean annual temperature (MAT), than did fine roots. Initial litter lignin content was the most important predictor of fine root decomposition, while lignin to nitrogen ratios, MAT, and mean annual precipitation were the most important predictors of coarse root decomposition. Our study emphasizes the necessity of separating fine roots and coarse roots when predicting the response of belowground C release to future climate changes.

Litter decomposition can have large impacts on biogeochemical cycling at local, regional, and global scales^1^. In terrestrial ecosystems, this degradation process recycles nutrients and is the source of large fluxes of CO_2_ into the atmosphere^2^,^3^,^4^,^5^. One noteworthy feature of litter decomposition is the pattern of decay constants (*k*-values) and their controlling factors. Several meta-analyses have summarized the factors controlling litter decomposition from leaf litter^2^,^6^, fine roots^4^, wood^7^, and comprehensive leaf, woody, and root debris^8^ at large spatial scales. In contrast, relatively little attention has been paid to exploring the patterns in coarse root decomposition in terrestrial ecosystems.

Root diameter is a key factor that governs root decomposition^9^ because it integrates both chemical and physical properties associated with root development^10^. Roots are commonly divided into fine and coarse root categories (defined by root diameters of less than or at least 2 mm, respectively) that are also distinguished by their functional roles^4^,^11^. They differ in morphological traits, such as specific root length and root tissue density, and nutrient (e.g. nitrogen [N] and phosphorus [P]) concentrations^12^,^13^. Nutrients, oxygen, and water are obtained by fine roots and their associated mycorrhizae, while coarse roots support the fine root network, deliver nutrients and water to shoots, and support the plant structure^14^,^15^,^16^.Fine roots represent a substantial proportion of net primary productivity^17^. Fine root decomposition is believed to represent a large carbon (C) cost to plants^18^ and to serve as a potential soil C source^19^. Fine roots track changes in aboveground phenology, soil temperature, and moisture and nutrient availability^20^, with consequent seasonal changes in biomass and distribution and high annual turnover rates^21^,^22^,^23^,^24^,^25^. In contrast, coarse roots often reflect the aboveground biomass. Tree size and age have been suggested as predictors of coarse root size^26^,^27^. With its slow turnover of C and nutrients, coarse root decomposition may be more important to long-term ecosystem productivity^28^,^29^,^30^. Although several studies have shown that fine and coarse roots differ markedly in their morphology, nutrient concentrations, functions, and decomposition mechanisms^31^,^32^,^33^,^34^, the global pattern between the decomposition of fine and coarse root is still largely unknown.

Both climate and initial litter quality have been previously recognized as major decomposition-controlling factors at large spatial scales^2^,^4^,^35^. Temperature and precipitation are closely related to the spatial variation of soil C release^36^. Forecasts of significant climate change have placed climate feedbacks, as reflected by changes in litter turnover, and thereby C stocks, high on the international research agenda^37^. Over the next century, mean annual temperature (MAT) is predicted to rise by 1.8–4.0 °C, while precipitation frequency and intensity are expected to change at both regional and global scales^38^. Litter quality is believed to explain the largest amount of variability in global-scale root decomposition^4^. Recent meta-analyses have also suggested that interspecific variation in leaf litter quality has stronger effects on litter decomposition^8^,^37^. Root tissue quality generally differs between fine and coarse roots^29^. For instance, initial N and P concentrations are generally higher in fine roots than coarse roots^12^,^13^. Differences in litter tissue quality may induce different decomposition responses to temperature. Because temperature sensitivity of litter decomposition is inversely proportional to substrate quality^39^,^40^,^41^, coarse root decomposition may have a higher dependence on temperature than that of fine roots. On the other hand, Silver and Miya (2001) have found that initial root calcium (Ca) content explains the largest extent of variability in root decomposition rates. Because of limited data sources at the time of their study, however, possible differences in the factors controlling fine and coarse root decomposition were not fully investigated. Studies on belowground root decomposition have focused mostly on fine roots, with very little attention paid to coarse roots^42^,^43^. Whether the patterns applicable to fine root decomposition hold for coarse roots is still not clear. Given the fact that coarse roots account for most root biomass (except in very young stands)^44^, an investigation of the factors controlling coarse root decomposition is of crucial importance.

To develop a more thorough understanding of the factors that control fine and coarse root decomposition and the response of these factors to climate change, we established a comprehensive dataset. The dataset comprised 530 observations from 71 sampling sites, and contained information on initial root chemistry, including N, P, lignin, Ca, C to N, and lignin to N ratios as well as climate variables (MAT and mean annual precipitation [MAP]). This dataset enabled investigation of two questions, namely determination of whether a common global pattern of decomposition rates exists between fine and coarse roots, and identification of the main controlling factor(s) for fine and coarse root decomposition. On the basis of our results, we formulated two tentative conclusions regarding these questions: first, decomposition rates of both fine and coarse roots decrease with increasing latitude, and second, coarse roots decompose globally more slowly than fine roots. In addition, coarse root decomposition was found to have a higher dependence on MAT than that of fine roots, and coarse root and fine root decompositions were revealed to differ in their responses to litter quality.

## Materials and methods

### Data source

Data on root decomposition were collected from the published database of Silver and Miya (2001) and from other published papers not included in earlier syntheses (Supplement Table 1). To access studies related to root decomposition, we searched the ISI Web of Knowledge using the keywords “root” and “decomposition”, “decay”, or “mass loss”. Several criteria were established for developing the database: (1) Root decay constants (*k*-values) should have been measured *in situ* so as to remove any potential effects from home-field advantage^45^; (2) the experimental data in the original paper should not have been designed for special purposes (e.g. fertilization, warming, or grazing); (3) *k*-values should have been estimated by the litterbag technique—the best available method, although not without limitations^46^, for generating large decomposition datasets^4^,^47^; (4) only *k*-values reported using a single exponential model in the original paper or that could be calculated from figures or tables were considered; (5) clear ancillary site information or study site latitude and longitude should be determinable from the site description using Global Gazetteer Version 2.1 ( http://www.fallingrain.com/world/). Because no significant effect of mesh size on root decomposition has been identified^4^, studies using different mesh sizes were not separated.

Climatic variables, specifically MAT and MAP, were obtained from the original papers. Although other research has suggested that actual evapotranspiration (AET) is a better climatic indicator for studying decomposition^2^, this information was rarely provided in most papers. In fact, AET cannot be directly acquired by instruments under field conditions, and is very sensitive to heterogeneity in time and space (such as soil type, rooting depths, and available soil moisture)^48^,^49^. In addition, reliable general models to evaluate AET are presently lacking. We therefore used MAT and MAP to represent climatic factors, as did previous studies^4^,^50^,^51^. In cases where the original MAT and MAP were not given, they were inferred from the WorldClim 1.4 database ( http://www.worldclim.org/)^52^. To test the accuracy of the inferred data, we also estimated the climatic variables reported in the original papers and compared their values to the observed ones. The results of this comparison indicated that both MAT (r^2^ = 0.86) and MAP (r^2^ = 0.92) could be accurately simulated (Fig. S1). While most papers included MAT and MAP information for only their study periods (mostly within a single year), our inferred MAT and MAP values were averaged over 50 years (1950–2000). Although the data source was not consistent, the inferred data nonetheless offered us reliable results (Fig. S1). In addition, monthly data for climatic factors were rarely given in the original paper; interannual variation was therefore not considered in our analysis. Furthermore, we assumed that variation in MAT and MAP was greater than that of interannual variation with respect to the geographic gradient.

We also compiled several indices of initial root chemistry that have been previously suggested to be important decomposition-controlling factors^4^,^53^: N concentration (mg g^−1^), C to N ratio, P concentration (mg g^−1^), lignin content (%), lignin to N ratio, and Ca concentration (mg g^−1^). At least one of these properties was either reported in each original paper or could be extracted from published graphs using the software program Originpro 7.5 (OriginLab, Northampton, MA, USA). A final comprehensive database was obtained that contained 530 observations from 71 global sampling sites (Supplement Table 1). In the database, MAT ranged from −3.8 to 28.2 °C, and MAP ranged from 90 to 5,050 mm. Latitudes of the collected data ranged from 5.3 to 62.6 °N or S.

### Data analysis

Fine and coarse roots were defined as having root diameters of less than or at least 2 mm, respectively^4^,^11^. Data from papers in which no specific root diameter was provided (except for some graminoid species which we assumed that the roots were in fine root category) or the criterion separating fine and coarse roots was not consistent with the above definition were not used for subsequent analysis. As a result, a total of 336 data points were analyzed, 273 from fine roots and 63 from coarse roots (Fig. S2). The data were further divided into those associated with low latitude areas (defined as ≤30 °N or S) and middle latitude areas (defined as >30 °N or S) to compare differences in *k-*values between fine and coarse roots using an independent-sample *t*-test. One-way analysis of variance was used to test for differences in initial root chemical parameters between fine and coarse roots. Simple linear regressions were performed to determine whether root decay constants were correlated with initial root chemistry or climatic variables. The *k-*values were log-transformed (base *e*) to meet normality and homogeneity assumptions for correlation and regression analyses. Parameters significantly correlated with the decay constant *k* in the simple linear regressions were subjected to multiple stepwise regression analysis to separate the effects of climate, initial root chemistry, or the combination of climate and root chemistry. We took this analysis method in the aims of excluding potential factors with small amount and insignificant effect on root decomposition. All statistical analyses were performed with a significance level of *P* < 0.05 using SPSS ver. 18.0 (SPSS Inc., Chicago, IL, USA).

## Results

### Initial root chemistry and decay constants

**Figure 1:**
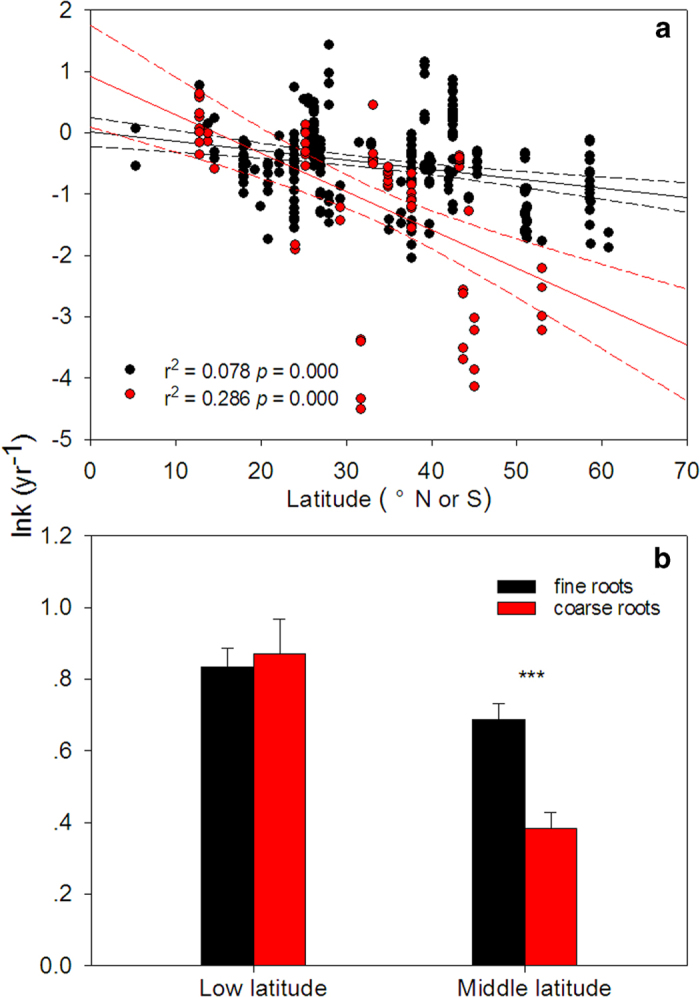
Pattern of fine and coarse root decomposition rates (ln *k*) with latitude (**a**) and comparison of root decomposition for fine and coarse roots between low and middle latitude areas. *** represents *t*-test significance at *p* < 0.001.

N concentrations were significantly higher in fine roots than in coarse roots (1.1–27.8 mg g^−1^ vs. 1.6–14.9 mg g^−1^). P concentrations and lignin contents did not significantly differ between fine and coarse roots (0.3–2.2 mg g^−1^ vs. 0.3–1.1 mg g^−1^ for P; 8–48% vs. 17–42% for lignin). Ca concentrations were significantly higher in coarse roots than in fine roots (7.8–31.7 mg g^−1^ vs. 1.6–23.3 mg g^−1^). Both fine and coarse root decay constant *k-*values decreased with latitude (Fig. 1a). Fine roots decomposed faster than coarse roots in middle latitude areas (Fig. 1b) or when all the data were combined (Table 1). In low latitude areas, however, no significant differences were found between fine and coarse roots (Fig. 1b). Among different life form species, *k*-values (year^−1^) followed the order graminoids (1.27 ± 0.12) > shrubs (1.02 ± 0.09) > broadleaf trees (0.71 ± 0.05) > conifers (0.41 ± 0.04). Species compositions for both fine and coarse root data were quite similar. Tree species (i.e. conifers and broadleaf trees) accounted for 70% and 66% of fine and coarse root data, respectively. Graminoids represented 28% and 20% of the total amount of fine and coarse root data. Only a small amount of data, especially in the case of fine roots, came from shrubs.

### Effects of climate and initial root chemistry on decomposition

**Figure 2:**
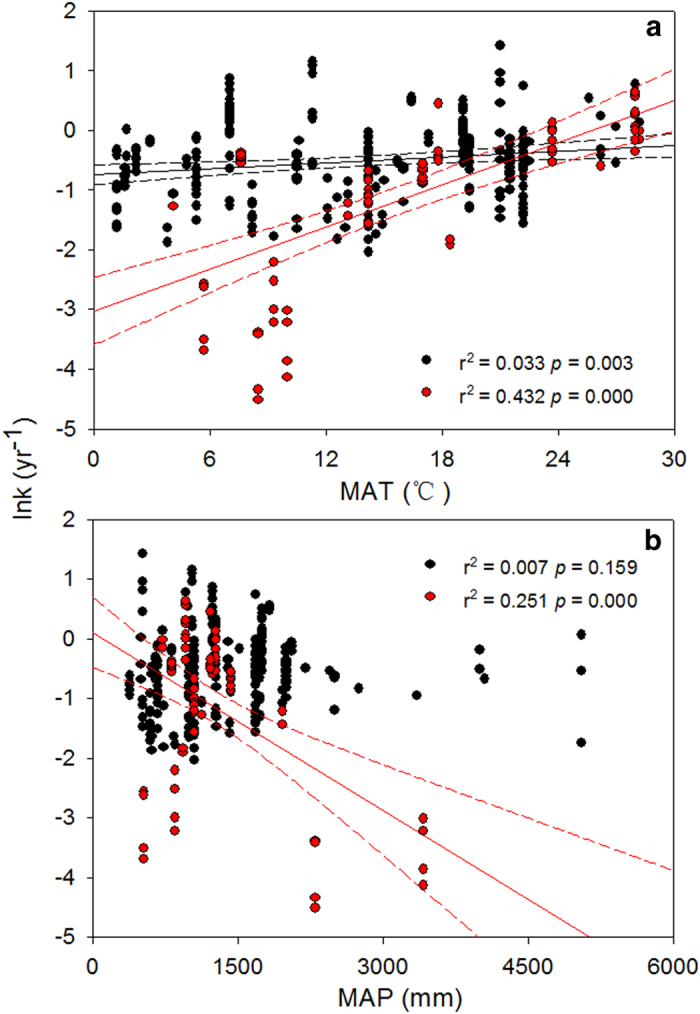
Simple linear regressions between decomposition rates (ln *k*) and mean annual temperature and mean annual precipitation between fine and coarse roots. The solid line represents a significant linear relationship, and the dashed line is the 95% confidence interval. Black and red dots represent fine and coarse roots, respectively.

Fine and coarse root decompositions differed in their responses to climate. MAT exerted a stronger effect on coarse roots than fine roots, explaining 43% of the variation in decay constant *k*-values in coarse roots and only 3% of the variation in fine roots (Fig. 2a). MAP was negatively correlated with the decay constants of coarse roots, explaining 25% of the variation in *k*-values (Fig. 2b). The combination of MAT and MAP explained 59% of the variation in coarse root decomposition (Table 2).

**Figure 3:**
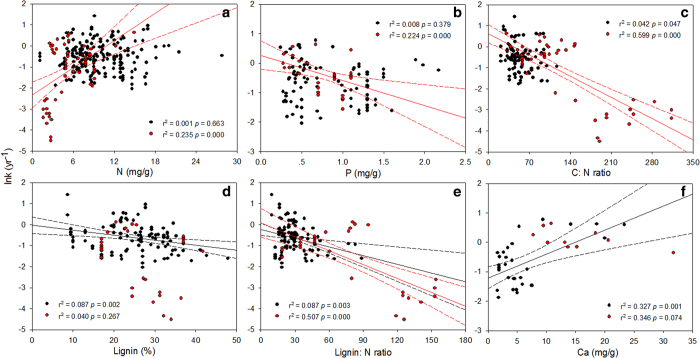
Simple linear regressions between decay constants (ln *k*) and initial root quality for nitrogen (N) (**a**), phosphorus (P) (**b**), carbon (C) to N ratio (**c**), lignin (**d**), lignin to N ratio (**e**), and calcium (Ca) (**f**). The solid line represents a significant linear relationship, and the dashed line is the 95% confidence interval. Black and red dots represent fine and coarse roots, respectively.

Fine and coarse root decomposition also differed in regard to response to initial root chemistry. According to the simple linear regression analysis, initial root N and P concentrations significantly influenced coarse root decomposition (Fig. 3a, b) but did not affect fine roots. Initial root C to N and lignin to N ratios explained 60% and 51%, respectively, of the variation in decay constants for coarse roots (Fig. 3c, e). Root Ca concentrations explained 33% of the variation in fine root decomposition (Fig. 3f). When multiple stepwise regression analysis was conducted, initial root lignin content was the best predictor, explaining 66% of the variation in fine root decomposition (Table 2). Lignin to N ratio was the major driver of coarse root decomposition, explaining 62% of the variability (Table 2).

When both climate and root chemistry were considered, the explanatory capability for coarse root decomposition increased to 86% (Table 2). For fine roots, however, the combination of climate and root chemistry did not improve prediction capacity (Table 2).

## Discussion

The research of Silver and Miya (2001) has suggested that root decomposition is regulated by major variations in tissue chemistry among root diameter classes. Because of the limited amount of data previously available, however, they were unable to directly compare differences between fine and coarse root decomposition. Based on their work, we further investigated the controlling factors between fine and coarse root decomposition and obtained new insights into global patterns of root decomposition rates and influencing factors.

First, we found that the major decomposition-controlling factors between fine and coarse roots differ among latitudes. Both fine and coarse roots decompose faster in low latitude areas than at middle latitudes. This pattern is most likely caused by the inverse relationship between latitude and temperature (r^2^ = 0.64, *p* = 0.001). In addition, values of root chemistry indices (e.g. N concentration, P concentration, and lignin content) in different latitudes are quite similar, providing more evidence that temperature promotes decomposition in low latitude areas. Other factors, such as soil C and N content, may also influence root decomposition rates^54^. Only a few studies have provided these data as background information, however, so this idea could not be readily tested. In specific latitude areas, fine and coarse root decomposition shows different responses to controlling factors. It is surprising that coarse roots decompose as fast as fine roots in low latitude areas. The similar decomposition rates between fine and coarse roots may be the result of different species compositions. In low latitude areas, fine root data was collected from graminoids, conifers, broadleaf trees, and shrubs, whereas coarse root observations were from broadleaf trees and shrubs. Conifers have lower decomposition rates than other species (0.41 for conifers vs. 1.27 for graminoids, 0.71 for broadleaf trees, and 1.02 for shrubs), and may consequently decrease calculated fine root decay rates. Another possible reason for the similar decomposition rates between fine and coarse roots may be the negative relationship between MAP and fine root decomposition (Fig. S3), with variations in precipitation pattern somehow counteracting the fast decomposition rates of fine roots. Because of the mixed effect of life form and climate-driven changes on decomposition rates, further work is needed to accurately determine the reason for the similarity of decomposition between coarse and fine roots.

In contrast to observations in low latitude areas, we found that coarse roots decompose much more slowly than fine roots (0.38 vs. 0.69 year^−1^) in middle latitude areas. Species compositions from fine and coarse roots are quite similar in this area, while root chemistry is different. Higher N concentration and less lignin content might increase fine root decomposition rates. In these areas, the differences in decomposition between fine and coarse roots may thus be mainly due to litter quality. Apart from differences in litter quality and climate, however, significant differences in soil microbial communities may exist between mid-latitude and low latitude areas^55^. The effects of such differences in soil microbial communities require further investigation.

Second, on a global scale, we observed that MAT has a stronger effect on the decomposition of coarse roots than fine roots (Fig. 2a). This relationship suggests that temperature-induced increases in coarse root decomposition may have more potential to exacerbate increasing atmospheric CO_2_ levels, thereby providing a positive feedback to global warming. Q_10_ values, an indicator of temperature sensitivity in root decomposition, were found to be higher in coarse than fine roots (3.24 vs. 1.18, respectively). The higher temperature sensitivity of coarse root decomposition will influence rates of ecosystem C sequestration in a warmer world^56^. Our observations that coarse roots have lower N concentrations and higher C to N ratios than fine roots (Table 1) are consistent with fundamental principles of enzyme kinetics and the C quality-temperature hypothesis that suggests that lower-quality C substrates are more sensitive to temperature^15^,^56^,^57^. Our study results therefore emphasize the importance of coarse roots in the global C cycle and indicate potential feedbacks to climate change.

Fine and coarse roots also differ in their response to MAP (Fig. 2). No significant correlation was found between decay constants of fine roots and MAP because of the contrasting effects of a positive relationship in middle latitude areas and a negative relationship in rainy low latitude regions (Fig. S3). The decrease in decay constant *k*-values with increasing MAP in high rainfall areas may be because high rainfall limits oxygen diffusion^58^ and increases soil leaching rates^59^, thereby leading to low nutrient and pH values that potentially reduce the activities of soil decomposers^60^. In coarse roots, the trend of decreasing decay constant *k*-values with increasing MAP may be attributable to the inverse relationship between litter quality and MAP. For instance, the increase in the lignin to N ratio (the main controlling factor for coarse root decomposition) with increasing MAP (Fig. S4) can indirectly impede coarse root decomposition. Our study results demonstrate that the decompositions of fine and coarse roots differ in their responses to climate, and thus emphasize the importance of root diameter classification to predict the response of root decomposition to global climate changes.

Finally, our survey revealed that fine and coarse roots differ in their response to initial root chemistry (Table 2). Ca concentrations in plant tissues are believed to limit root decomposition at the global scale^4^, but responses between fine and coarse roots have not been differentiated. Our data indicate that initial lignin content is the major factor controlling fine root decomposition rate and that lignin to N ratio is the major factor for coarse roots (Table 2). Although the simple linear regression analysis suggested that root Ca is the best predictor of fine root decomposition (Fig. 3f), root lignin was found to be the major predictor of fine root decomposition when multiple stepwise regression was used (Table 2).

Decomposition does not take place (*k* < 0) in tissues with initial lignin to N ratios much greater than 29^61^. The microorganisms involved in litter decomposition in these instances have to rely largely on the original N stocks of the decomposing tissues. If relatively large amounts of N are available to the microorganisms involved in litter decomposition, then the initial N content of the litter may not exert as great an influence on decomposition rates; the lignin content of the litter may thus become more important in the determination of decomposition rates^62^. In our study, fine roots had significantly higher initial N concentrations than coarse roots; initial N concentrations are therefore unlikely to be a limiting factor for microbial decomposition of fine roots. For coarse roots with lower initial N concentrations, in contrast, both initial lignin and N concentrations may be important for determination of decomposition rates. Lignin controls decomposition rates through its resistance to enzymatic attack, as well as through physical interference with the decay of other cell wall fractions^63^. Recent research, however, has suggested that higher-order fine roots (containing more lignin) decompose faster than lower-order fine roots regardless of lignin content 64,65,66. Because our study involved different species compositions varying in decomposition rates, our results may be not comparable with those from studies using the same species to observe the influence of lignin on decomposition rate.

Although our results provide new insights into root decomposition, there are still some limitations. For instance, although the relationship between decomposition constant and main controlling factors were statistically significant based on *p*-values, the r^2^ values were mostly very low (indicating quite limited future predictive power). The discovery of trends in root decay constants related to climate and root quality would thus be difficult. Consequently, our results still do not provide an accurate global pattern. The absence of a revealed global pattern calls for further investigation. In addition, we treated every root chemistry variable as identical, but sample sizes varied tremendously. Because there are inadequate numbers of studies with complete root chemical parameters, the different sample sizes used from different studies may have induced some uncertainties into the analysis. Furthermore, we only considered the effect of climate and initial litter quality on fine and coarse root decomposition rates. If global climate changes cause substantial shifts in plant community composition, these interacting biotic factors might have greater impacts on decomposition and biogeochemical cycles than the single factor of rising atmospheric temperature^67^,^68^,^69^. Moreover, the models of decomposition based on climate and litter chemistry in our study also ignored the potential influence of microbial community structure and soil fauna on litter decomposition rates^33^,^70^,^71^,^72^,^73^,^74^. Finally, our conclusions, which are based on short-term decomposition, should be treated with great caution: the factors that best correlate with rates of early decay are often not the same as those related to long-term decay^1^.

## Conclusions

We conducted a comprehensive global survey on the control of decomposition rates between fine and coarse roots. We found that coarse roots decompose as quickly as fine roots in low latitude areas, which challenges the traditional viewpoint that coarse roots should display slow delivery of soil C and nutrients. A higher dependence of coarse root decomposition rates on climate, especially temperature, implies their key role in global C cycling and climate change response. Coarse root decomposition also differed from fine root decomposition in its response to initial litter quality. Our results suggest the classification of root diameter is important to predict the responses of belowground C release to future global climate change.

## Additional Information

**How to cite this article**: Zhang, X. and Wang, W. The decomposition of fine and coarse roots: global patterns and their controlling factors. *Sci. Rep.* 5, 9440; doi: 10.1038/srep09940 (2015).

## Supplementary Material

Supplementary Information Supplementary Figures 1-5, Supplementary Materials

Supplementary Dataset 1

## Figures and Tables

**Table 1:** 

**Parameters**	**<2 mm**	**n**	**≥2 mm**	**n**
*k*-value***	0.75 (0.04)	266	0.53 (0.05)	71
nitrogen (mg/g)***	9.69 (0.29)	193	5.76 (0.34)	68
carbon to nitrogen ratio***	55.11 (2.32)	95	115.80 (9.73)	56
phosphorus (mg/g)	0.84 (0.05)	95	0.79 (0.05)	34
nitrogen to phosphorus ratio	12.73 (0.89)	95	10.19 (0.99)	34
lignin (%)	24.96 (0.82)	104	27.50 (1.44)	33
lignin to nitrogen ratio***	35.27 (3.76)	104	73.65 (7.64)	33
calcium (mg/g)***	6.13 (1.03)	30	15.09 (2.24)	10

Root decay constants *k* and initial chemistry of fine and coarse roots. Data are presented as means (SE). n stands for the number of samples. *** represents *t*-test significance at *p* < 0.001.

**Table 2:** 

	**Regression equation**	**r**^**2**^	***p***	**n**
Climatic variables only				
fine roots	ln*k* = −0.742 + 0.005 MAT	0.03	0.003	266
coarse roots	ln*k* = −1.816 + 0.106 MAT + 0.000 MAP	0.59	0.000	71
				
Root initial chemistry variables only				
fine roots	ln*k* = 1.342–0.070 Lignin	0.66	0.000	17
coarse roots	ln*k* = 1.007–0.037 Lignin/N	0.62	0.000	25
				
Combined climate and root initial chemistry				
fine roots	ln*k* = −0.425 + 0.023 MAT—0.021 Lignin	0.13	0.002	104
coarse roots	ln*k* = −2.384 + 0.149 MAT + 0.000 MAP—0.01 Lignin/N	0.86	0.000	33

Multiple stepwise regressions of climate, initial root chemistry, and the interaction of climate and chemistry on fine and coarse root decay constants *k*. r^2^ and n represent the determinant index and sample numbers, respectively. Regressions are significant at *p*  < 0.05.
