# Supplementary material for: The decomposition of fine and coarse roots: their global patterns and controlling factors
Source: Sci Rep. 2015 May 5;5:9940. doi: 10.1038/srep09940 (PMC4649993; doi:10.1038/srep09940)
Supplement: Supplementary Information — Supplementary Figures 1-5, Supplementary Materials [file srep09940-s1.doc]

**Title page**

**The Decomposition of Fine and Coarse Roots: Their Global Patterns and Controlling Factors**

Authors,

**Xinyue Zhang^1,2^, Wei Wang^1^**

^1^ Department of Ecology, College of Urban and Environmental Sciences, Peking University, Beijing 100871, CHINA

Email address, scarlet.yue@gmail.com

Postal address, College of Urban and Environment Science, Room 3648, Yifu Building 2#, Yiheyuan Road 5#, Haidian District, Beijing, China 100871

^2^ Shenzhen Graduate School, Peking University, Shenzhen 518055, China

Email address, wangw@urban.pku.edu.cn

**Supplement materials**

Figure S1 Comparison of mean annual temperature (MAT) (a) and mean annual precipitation (MAP) (b) between observed values and inferred ones from global climate database. Linear regression equations between observed and inferred values were *Y* = 0.97*X* +0.84 for MAT (r^2^ = 0.86, *p* < 0.001) and *Y* = 0.95*X* +102.82 (r^2^ = 0.92, *p* < 0.001)

Figure S2 Geographic distribution of the sites contained in the database, preparing by R 3.1.3 with the package of maps. Green triangles represent sites without clear indication of root diameter. Red, blue and orange circles represent the sites of fine roots, coarse roots and both of them, respectively.

Figure S3 The relationship between decay constant *k* value and climate for mid-latitude area and low-latitude area

Figure S4 Positive relationship between initial root Ca concentrations and MAT

Figure S5 Positive relationship between initial root lignin: N ratios and MAP


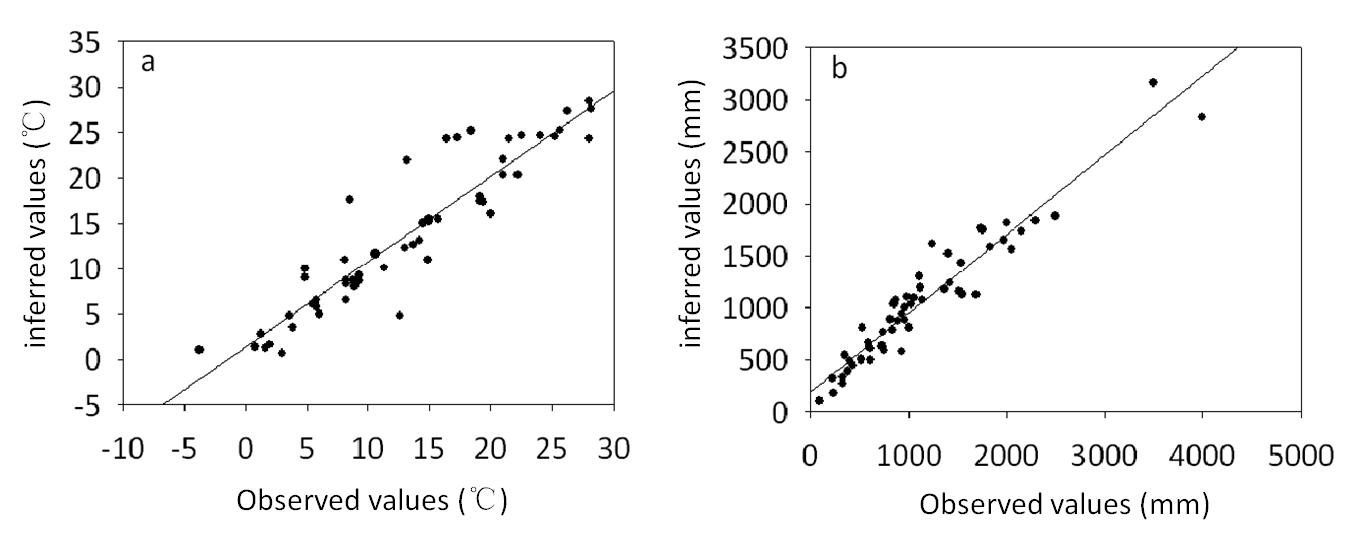


Figure S1

Figure S2


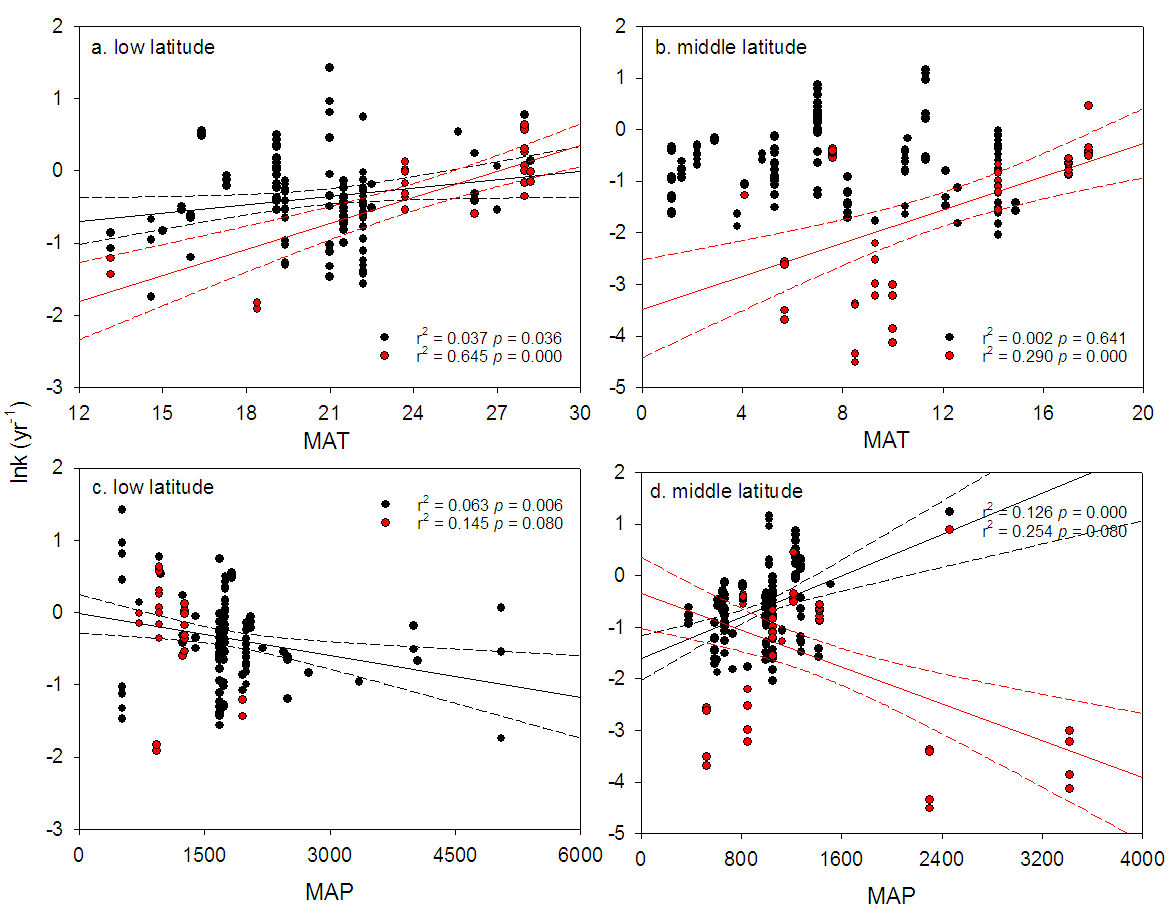


Figure S3


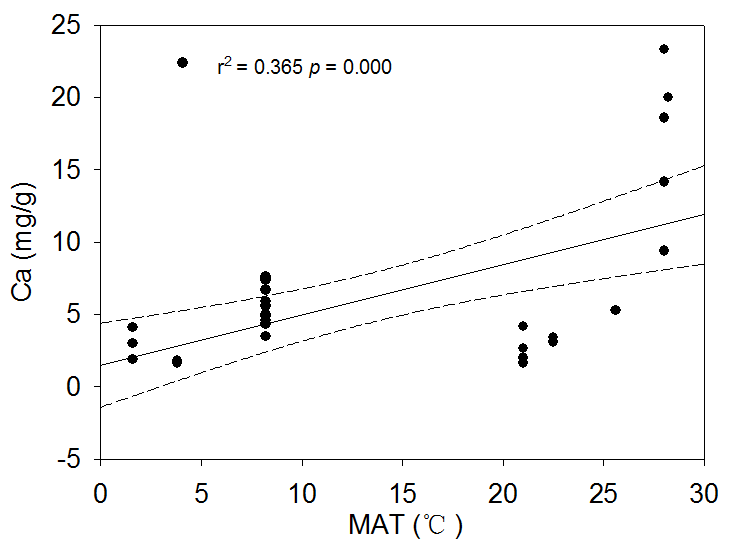


Figure S4


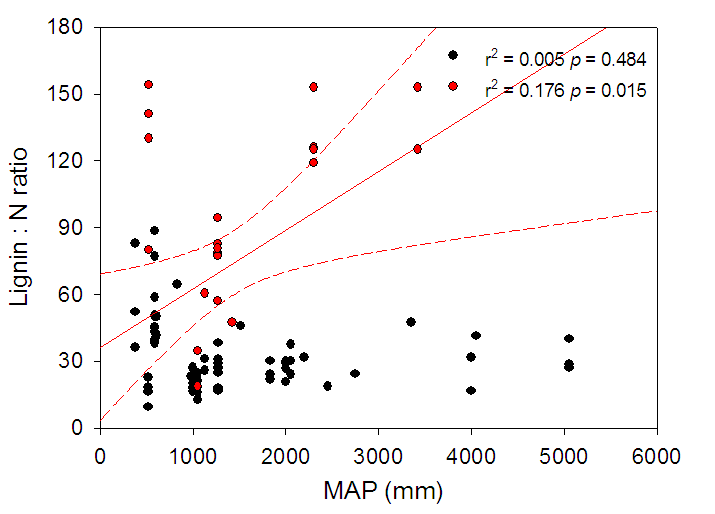


Figure S5
